# The Top 100 Most-Cited Articles on the Treatment of Lymphedema

**DOI:** 10.7759/cureus.50887

**Published:** 2023-12-21

**Authors:** Abdullah M Alahwal, Hattan Aljaaly

**Affiliations:** 1 Department of Otorhinolaryngology, Faculty of Medicine, King Abdulaziz University, Jeddah, SAU; 2 Department of Plastic Surgery, Faculty of Medicine, King Abdulaziz University, Jeddah, SAU

**Keywords:** publications, research, articles, citation, lymphedema

## Abstract

A multitude of articles have been published on lymphedema management. We aim to identify the 100 most-cited articles on the management of lymphedema and perform a bibliometric analysis. In July 2023, a title-specific search was made on the Scopus database using “lymphedema” as the primary search term. The top 100 most-cited articles were reviewed. The top 100 most-cited articles on lymphedema received a mean citation of 81.7 ± 71.9 per article (range of 11.0 to 420.0). The publication dates ranged from 1977 to 2015. Most of the articles were original (63.0%), interventional studies (35.0%), randomized controlled trials (RCTs) (31.0%), and systematic reviews (32.0%). The largest number of articles (31) were found between 2007 and 2011. The top 10 articles’ citation counts ranged from 164 to 420 (mean of 244.7 ± 83.9 citations). Five of these 10 articles were published between the years 1990 and 2000. Twenty-five countries contributed to the 100 most-cited articles. The United States produced the most number of articles (n = 32), followed by Italy (n = 11), Sweden, and Turkey, with seven articles each. Four of the top 10 articles were RCTs; the remaining six were systematic, retrospective, and prospective studies. The New England Journal of Medicine published two of these top 10 articles. Retrospective studies had the highest mean citation with 196.5, followed by RCTs with 100.9. We identified the 100 most-cited articles that depict the advancement in treatment methods for lymphedema. This extensive information directory can be an excellent source for further research.

## Introduction and background

Lymphedema is a “chronic, progressive condition in which extra lymph fluid accumulates in the subcutaneous tissue, causing it to swell due to inadequate lymphatic function” [1, 2]. It can occur in various body parts, particularly in the extremities, when parts of the lymphatic system become blocked, damaged, or removed [2]. Lymphatic channels can become blocked or damaged, resulting in an imbalance between capillary filtration and lymphatic drainage, causing extremity swelling, heaviness, and/or pitting [3]. The diagnosis of lymphedema is based on history and physical examination and confirmed by diagnostic methods, including lymphoscintigraphy [3, 4].

Several reports have associated lymphedema with cancer, specifically breast and gynecological cancers. It was shown that lower-extremity edema occurred in 23% of patients with endometrial cancer [5]. A study reported that lymphedema of the lower limbs occurs more frequently as an adverse side effect of lymphadenectomy in hysterectomy and bilateral salpingo-oophorectomy [6]. Furthermore, patients who had radiotherapy, a body mass index greater than 25 kg/m2, or lymph node dissection had a greater risk of developing lower limb lymphedema after treatment for gynecologic neoplasms [7, 8]. Patients who have undergone a lymph node biopsy for breast cancer also have a clinically relevant risk of developing lymphedema [8]. Lymphedema also develops late in patients with head and neck cancer [9].

Early referral for treatment should be considered when lymphedema is noted [9]. Patients at risk for lymphedema (particularly those with gynecologic or breast cancer) should be instructed to detect early signs and symptoms, as there are several cases of symptomatic but undiagnosed lymphedema in this population [10, 11]. Management is directed towards decreasing the extremity swelling while trying to maintain the reduction in extremity swelling, preventing complications, and improving function and overall psychological well-being [4]. Many studies have been conducted on lymphedema; most have looked into the disease setting when lymphedema is encountered [12].

## Review

Methods

in July 2023, we performed a title-specific search of the Scopus database using the query term “lymphedema” to identify highly cited articles without restricting publication dates. The retrieved list of articles on lymphedema was sorted according to the number of citations in descending order. The top 100 articles with the highest number of citations were obtained and reviewed. A review of the abstract/article and data, which included the title of the article, year of publication, first and last authors, study design, country of origin, name of the journal, type of article, treatment methods, specific techniques tackled, and outcomes of treatment, were extracted. Bibliometric analysis was performed using the Scimago journal impact factor.

Results

The top 100 most-cited articles on lymphedema received a mean citation of 81.7 ± 71.7 per article (range of 11.0 to 420.0). The publication dates ranged from 1977 to 2015. Table 1 shows the characteristic profile of the top 100 most cited articles on lymphedema [3, 6-15].

**Table 1:** Characteristic profile of the top 100 most-cited articles on lymphedema [3, 6–15]

Journal characteristics	Mean ± SD (range)	n (%)
Number of citations per article	81.7 ± 71.9 (11.0 to 420.0)	
Journal impact factor	7.44 ± 26.48 (0.271 to 244.5)	
Country of origin (with more than two articles)		
United States		32 (32.0%)
Italy		11 (11.0%)
Sweden		7 (7.0%)
Turkey		7 (7.0%)
United Kingdom		6 (6.0%)
Australia		6 (6.0%)
Brazil		3 (3.0%)
Japan		3 (3.0%)
Netherlands		3 (3.0%)
Types of articles		
Original article		63 (63.0%)
Review article		32 (32.0%)
Conference paper		7 (7.0%)
Audit / Report		2 (2.0%)
Study designs		
Interventional study		35 (35.0%)
Prospective randomized trials		31 (31.0%)
Retrospective cross-sectional		2 (2.0%)
Systematic reviews		32 (32.0%)

Most of the articles were original articles (n = 63, 63.0%), and studies were interventional (35.0%), randomized controlled trials (RCT) (31.0%), and systematic reviews (32.0%).

**Figure 1:**
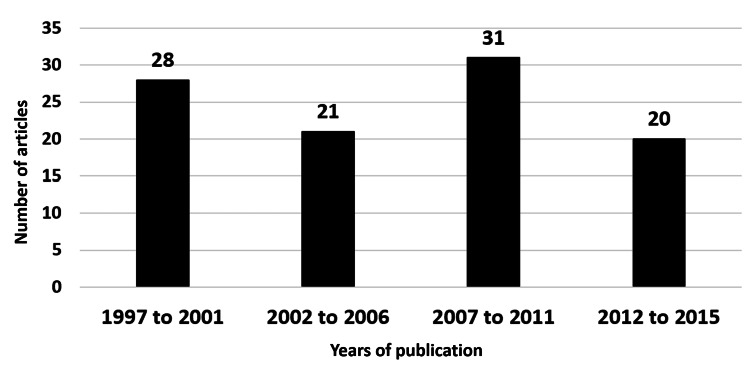
Number of articles published per five-year intervals

According to the publication trends by five-year intervals, the greatest number of most-cited articles published was between 2007 and 2011, with 31 articles, followed by 1997 to 2001, with 28 articles. Figure 1 presents the most frequently cited article, a systematic review article authored by Erickson et al. (USA) entitled “Arm edema in breast cancer patients," which had 420 citations and was published in the Journal of the National Cancer Institute (2001) which had an impact factor of 11.238 [13].

An analysis of the top 10 articles showed that their citation counts ranged from 164 to 420 [13-22]. Five articles were published between 1990 and 2000, and the other five were published between 2001 and 2009. Five articles were from the United States, and the other five were from France, Australia, Canada, Denmark, and Germany. Four articles were RCTs, three were systematic reviews, and the remaining three were retrospective and prospective studies. The New England Journal of Medicine published two of these top 10 articles (Table 2).

**Table 2:** Top 10 most-cited articles on lymphedema

Rank	No. of citations	First author	Last author	Title	Type of article/study	Year	Journal	Country
1	420	Erickson VS	Kahn KL	Arm edema in breast cancer patients [13]	Review/Systematic review	2001	Journal of the National Cancer Institute	USA
2	344	Schmitz KH	Greene QP	Weight lifting in women with breast-cancer-related lymphedema [14]	Original/Randomized trial	2009	New England Journal of Medicine,	USA
3	296	Becker C	Hidden G	Postmastectomy lymphedema: Long-term results following microsurgical lymph node transplantation [15]	Review/Retrospective cross-sectional	2006	Annals of Surgery	France
4	234	Ko DSC	Cosimi AB	Effective treatment of lymphedema of the extremities [16]	Original/Prospective cohort	1998	Archives of Surgery	USA
5	229	Casley-Smith JR	Piller NB	Treatment of Lymphedema of the Arms and Legs with 5,6-Benzo-[alpha]-pyrone [17]	Original/Randomized trial	1993	New England Journal of Medicine	Australia
6	214	Harris SR	Levine M	Clinical practice guidelines for the care and treatment of breast cancer: 11. Lymphedema [18]	Review/Systematic review	2001	CMAJ	Canada
7	199	Petrek JA	Smith RA	Lymphedema: Current issues in research and management [19]	Review/Systematic review	2000	Ca-A Cancer Journal for Clinicians	USA
8	181	Andersen L	Andersen J	Treatment of breast-cancer-related lymphedema with or without manual lymphatic drainage: A randomized study [20]	Conference/Randomized trial	2000	Acta Oncologica	Denmark
9	166	Szuba A	Rockson SG	Decongestive lymphatic therapy for patients with breast carcinoma-associated lymphedema: A randomized, prospective study of a role for adjunctive intermittent pneumatic compression [21]	Original/Randomized trial	2002	Cancer	USA
10	164	Baumeister RG	Siuda S	Treatment of lymphedemas by microsurgical lymphatic grafting: What is proved? [22]	Original/Retrospective cross-sectional	1990	Plastic and Reconstructive Surgery	Germany

**Figure 2:**
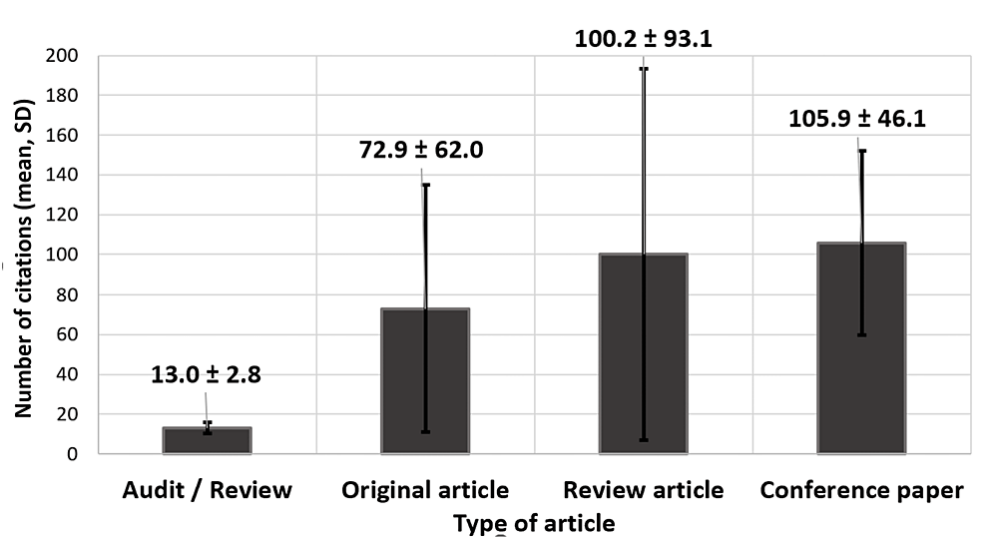
Mean (± SD) citation according to type of paper of the top 100 most-cited articles on lymphedema

The seven conference articles had the highest mean citation (105.9 ± 46.1), followed by review articles (n = 28) with a mean citation of 100.2 ± 93.1 (Figure 2).

**Figure 3:**
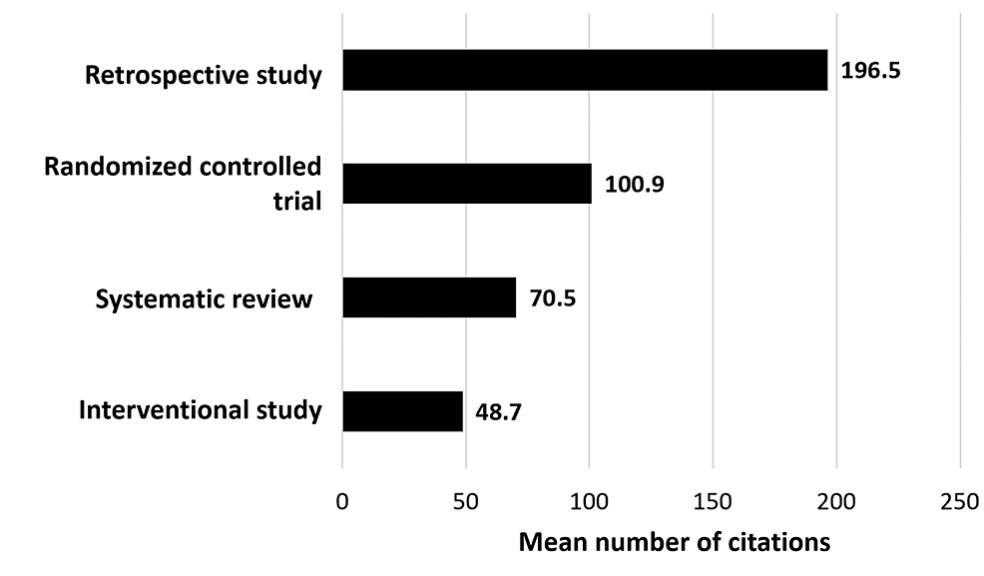
The mean number of citations according to the different study designs of the top 100 most-cited articles on lymphedema

According to the type of study (study design), retrospective studies had the highest mean citation with 196.5, followed by RCTs with 100.9 (Figure 3).

**Figure 4:**
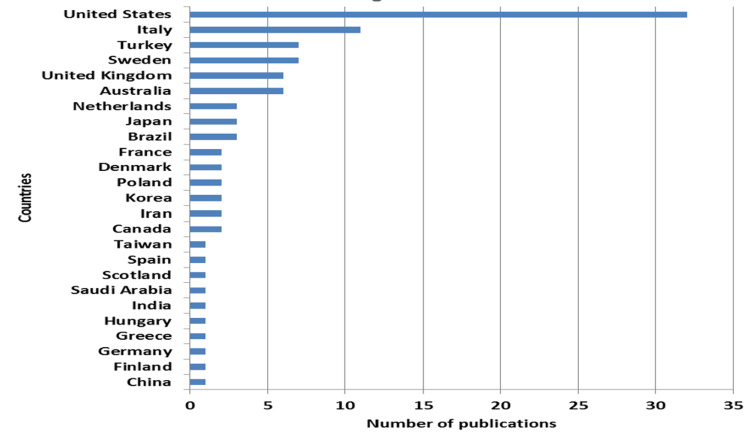
Number of publications per country of origin

Twenty-five countries contributed to the top 100 most-cited articles. The United States produced the most significant number of cited articles with 32 articles, followed by Italy with 11, and Sweden and Turkey with seven articles each (Table 1, Figure 4). 

Discussion

The citation analysis of significant literature on a particular subject matter in medicine plays a vital role in the progression and development of our understanding and epidemiology of diseases and their management and treatment. Several kinds of literature have been expounded on citation analysis in the fields of urology, radiology, emergency medicine, plastic surgery, neurosurgery, rehabilitation medicine, orthopedics, cardiovascular medicine, and many more [23-30]. However, an extensive literature search revealed no citation analysis of the most cited articles on lymphedema treatment.

Citation analysis is regarded as a method to measure the importance of an article or publication based on how many times the author, article, or publication has been cited and referred to by other works or authors. The Scopus database, which has 16,500 journals covering 19 million records going back to 1996, is by far the most user-friendly search interface, except that its citation tracking is limited to the relatively narrow period of around 1996 [31].

This article shows that the top 100 most-cited articles on lymphedema were published in 53 different journals, with 12 published in Lymphology, seven in Microsurgery, five in Plastic and Reconstructive Surgery, five in Cancer, and five in the Breast Cancer Journal. The remaining 66 articles were published in other journals of various subspecialties, from oncology to rehabilitation medicine. This shows that research in lymphedema and its readership interests vary widely across different medical specialties and subspecialties. Furthermore, most of the top most-cited articles on lymphedema have focused on interventional studies since 1997, in contrast to retrospective studies, which were not most cited from 2007 onwards. Randomized controlled studies on lymphedema increased to 30% of the most-cited articles from 2012 onwards. This means that researchers are now more interested in articles tackling comparative treatment, research articles with statistical reliability, and, of course, the publishability of the article [32].

The most cited article in our top 100 list was by Erickson et al. on arm edema in breast cancer patients [13]. It is a systematic review article published in the Journal of the National Cancer Institute on January 17, 2001. The article summarized research articles on the incidence, prevalence, risk factors, prevention, diagnosis, and management of arm edema related to breast cancer. The increasing prevalence of breast cancer and the search for management strategies, including the high morbidity and mortality of patients with breast cancer, may bring about a large number of citations for this paper. Breast cancer remains the leading cause of death among women worldwide, with 232,340 new cases and 39,620 deaths in the USA alone in 2013 [33]. Its management involves a multidisciplinary approach, including oncologists, internists, therapists, psychologists, and many more.

The second most cited article was in the New England Journal of Medicine titled “Weight lifting in women with breast-cancer-related lymphedema," authored by Schmitz et al. and published on August 13, 2009 [14]. This article outlined the effects of twice-weekly progressive weight lifting on the change in the arm and hand swelling of breast cancer survivors with stale lymphedema of the arm. This study showed that weightlifting did not significantly affect arm and hand swelling. However, the authors found a lower incidence of lymphedema exacerbations, reduced symptoms, and increased strength. Nevertheless, even though this study is not the most cited article on lymphedema, its average citations since its publication in 2009 are around 31.2, compared to Erickson’s article, with 22.1 average citations per year. Furthermore, this article was published in a journal with an impact factor of 79.258, translating into its vast readership and coverage.

The third most-cited article was the 2006 article by Becker et al. entitled “Postmastectomy lymphedema: long-term results following microsurgical lymph node transplantation," published in the Annals of Surgery, a journal with an impact factor of 9.203 [15]. This article evaluated the long-term effect of microsurgical lymph node transplantation on 24 breast cancer patients with lymphedema. Despite the successful outcome of their intervention, this study has cited an average of 21.1 citations per year since it was published. Another noteworthy article in our top 100 most-cited list is the systematic review entitled “Lymphedema: current issues in research and management” by Petrek et al. in 2000; it has been cited 199 times since its publication, and it was the only article that was published in a very high impact journal, Ca-A Cancer Journal for Clinicians, with an impact factor of 244.585 [19]. This article extensively tackled lymphedema's anatomical and physiological aspects, including the diagnosis and treatment of lymphedema as of 2000. However, since the publication of this article, it has cited an average of 10.4 citations per year as of this writing and received fewer citations in the last 10 years. This was probably due to the changing trends in diagnosing and treating patients with lymphedema.

The United States produced almost one-third of the top 100 most-cited articles on lymphedema. It is not surprising for the United States to remain the scientific powerhouse compared to other countries because of the immense support and investments in science and technology that the US government and private institutions poured in; the United States spent around US$ 500 billion in 2015, leading other countries in terms of budget and spending for research and development [34]. Despite China’s overtaking the United States in the volume of scientific publications in 2016 (China: 426,000 studies versus USA: 409,000 studies), studies from the United States still receive wide readership and publications in high-impact journals [34].

Another country worthy of mentioning is the sole article from Saudi Arabia, ranked 99th of the top 100 most-cited articles on lymphedema, which was published in the Journal of Physical Therapy Science in 2015 by Buragadda et al. from the Rehabilitation Sciences of King Saud University in Riyadh, Saudi Arabia. The study was a two-arm randomized study using post-mastectomy patients (conventional treatment using manual lymphatic drainage, low elastic compression garments, glenohumeral mobilization, and deep breathing exercises versus a decongestive therapy group who received complete decongestive therapy with a trained physiotherapist along with the conventional treatment for six weeks), where they measured arm circumference and arm function [35]. Although this article was published in a journal with a 0.271 impact factor, it has received 16 citations since it was published. This translates to the fact that Saudi Arabia is now emerging as a country that publishes quality research in the Middle East.

Although we were able to analyze the top 100 most-cited articles on lymphedema in this report, we could not further our analysis into subcategories. Furthermore, the sourcing of our data (Scopus) has limited complete bibliometric data for articles published before 1996. Therefore, before this date, citations may have yet to be noticed or under-recorded.

## Conclusions

We identified the top 100 most-cited articles on lymphedema using the Scopus database. We have highlighted the critical contributions of authors from various specialties. A large proportion of the most cited articles were mainly systematic reviews, interventional studies, and RCTs. These cited articles may guide future research and serve as an educational guide for trainees and authors. We also noticed that in recent years, the literature looking at lymphedema treatment has been limited when compared to other more popular topics, such as research related to facioplasty and breast augmentation surgery.
